# Adaptive simulation of 3D thermometry maps for interventional MR-guided tumor ablation using Pennes’ bioheat equation and isotherms

**DOI:** 10.1038/s41598-022-24911-1

**Published:** 2022-11-27

**Authors:** Julian Alpers, Maximilian Rötzer, Marcel Gutberlet, Frank Wacker, Bennet Hensen, Christian Hansen

**Affiliations:** 1 Otto-von-Guericke University, Faculty of Computer Science, Magdeburg, 39106 Germany; 2 Hannover Medical School, Institute for Diagnostic and Interventional Radiology, Hannover, 30625 Germany; 3 Otto-von-Guericke University, Research Campus STIMULATE, Magdeburg, 39106 Germany

**Keywords:** Cancer therapy, Computational science, Computer science, Software

## Abstract

Minimally-invasive thermal ablation procedures have become clinically accepted treatment options for tumors and metastases. Continuous and reliable monitoring of volumetric heat distribution promises to be an important condition for successful outcomes. In this work, an adaptive bioheat transfer simulation of 3D thermometry maps is presented. Pennes’ equation model is updated according to temperature maps generated by uniformly distributed 2D MR phase images rotated around the main axis of the applicator. The volumetric heat diffusion and the resulting shape of the ablation zone can be modelled accurately without introducing a specific heat source term. Filtering the temperature maps by extracting isotherms reduces artefacts and noise, compresses information of the measured data and adds physical a priori knowledge. The inverse heat transfer for estimating values of the simulated tissue and heating parameters is done by reducing the sum squared error between these isotherms and the 3D simulation. The approach is evaluated on data sets consisting of 13 ex vivo bio protein phantoms, including six perfusion phantoms with simulated heat sink effects. Results show an overall average Dice score of 0.89 ± 0.04 (SEM < 0.01). The optimization of the parameters takes 1.05 ± 0.26 s for each acquired image. Future steps should consider the local optimization of the simulation parameters instead of a global one to better detect heat sinks without a priori knowledge. In addition, the use of a proper Kalman filter might increase robustness and accuracy if combined with our method.

## Introduction

Volumetric monitoring of the heat distribution during minimally-invasive thermal ablation using radiofrequency, high focused ultrasound and microwave is an urgent clinical need. Magnetic resonance temperature imaging may be used to guide the radiologist during these kinds of interventions. Here, the 2D temperature maps can be computed based on the acquired phase images using the proton resonance frequency shift (PRFS) method^1^. To completely treat the tumor and prevent a recurrence, it is of greatest importance to destroy not only the malignant cells but also to provide a sufficient safety margin. This minimum ablative margin (MAM) is said to be one of the few predictors for local tumor recurrence. Laimer et al.^2^ claim that an increase of the MAM by one millimeter can decrease the risk of a local tumor recurrence by 30%. Nonetheless, the acquisition of 3D thermometry maps is accompanied by complex problems, like motion of organs due to the patient’s breathing^3^ and the restricted acquisition time during intervention. In our previous work^4^, we introduced a new image acquisition protocol by rotating a common 2D gradient recalled echo (GRE) sequence around the applicators main axis. This setup allows for a visual observation of the whole heat profile at any given time point. Nonetheless, our initial conventional reconstruction approach was susceptible to artifacts and MR inhomogeneities. In addition, the temporal resolution relied heavily on the duration of the image acquisition, resulting in delays of up to 6 s.

### Contribution

In this work, we introduce a new method for volumetric heat map generation which can be applied during minimally-invasive tumor ablation. Pennes’ BHTE is updated based on 2D thermometry maps acquired during ablation. Because these maps are rotated around the applicator’s main axis, the full heat profile is always visible, and the simulation is not restricted to a specific heat source term and can be applied to all ablation techniques. The accuracy of the initial parameters is negligible and optimized by minimizing the sum squared error between isotherms extracted from the 2D maps and the current 3D simulation. Evaluation shows promising results of up to $0.88\pm 0.04$ similarity to a manually extracted ground truth, while also being robust towards outliers and applicable to a wide range of clinical setups.

## Related work

Johnson and Saidel^5^ began research in the field of interventional and adaptive simulation in 2002 by analyzing 3D simulation for thermal processes and conducting one of the first theoretical studies. Based on this work, they and other researchers^6,7^ came to the same two conclusions. First, a continuous simulation of the heat distribution is able to increase the spatial resolution of a volumetric ablation monitoring. Second, a biological heat model is capable of aiding the real-time process of interventional radiology. A summary of the related work can be seen in Table 1.

Funetes et al^8^ show that it is possible to reconstruct missing data using the Kalman filter. In their work, they removed data from the images of an MRI-assisted thermal therapy, which were replaced by the BHTE modeled values. With consecutive data corruption below 10 sec, successful recovery was possible. Unfortunately, their approach was not meant to be used for interventional simulations of ablation. The focus of Senneville and Roujol’s group^9^ was to improve the accuracy and reliability of MRI thermometry data with respect to the influence of stochastic noise. This is because the calculation of the thermal dose for necrosis determination is highly error-prone at low SNR due to the exponential dependence on temperature.

**Table 1:** Summary of the related work.

	Temperature measurement	Treatment	Optimization	Parameter	Model	Discretization	Time [s]
^9^	MRI	HIFU	AEKF	Covariance of the Process noise *Q*	BHTE	Rayleigh /Fourier	–
^10^	MRI	HIFU	Binary feedback control	–	–	–	–
^11^	MRI	FUS	Rearrangement of the algebraic expression	*D* and $\alpha$	BHTE	Fourier	180-300
^12^	MRI	HIFU	PID controller	–	BHTE	Rayleigh /Fourier	–
^13^	MRI	FUS	Method of least squares	Equation parameter	Simple heat model	–	1
^14^	Sensors	Cryo	Levenberg-Marquardt	$\omega$ and *k*	BHTE	FEM	30
^15^	Sensors	RFA	Limited, nonlinear optimization function	$\omega$ and *k*	BHTE	SAR /CAD-software	–
^16^	MRI	LITT	Adjoint-Newton function	$\omega$ and *k* (non-linear)	BHTE	SAR / FEM	9
This work	MRI	MWA	Levenberg-Marquardt	*D* and $T_{max}$	BHTE	FDM	1.05±0.26

AEKF, adaptive extended Kalman filter; *D*, thermal diffusivity; $\alpha$, energy absorption rate; $\omega$, perfusion; *k*, thermal conductivity; FEM, finite elements method; SAR, specific absorption rate.

Another way to tackle this problem is through the use of control algorithms to determine the value of one parameter. A rather simple control algorithm has been developed by Enholm et al.^10^. Their aim was to adjust the duration of irradiation during a HIFU intervention. Orthogonal to a focused ultrasound beam, concentric circles were used to set temperature limits to which the ablation could maximally reach. These values were determined by a pre-interventional simulation in which the optimal irradiation was calculated. Temperature-sensitive MRI data using the PRFS method provide feedback on whether the voxels lying on the circle have reached the target temperature or thermal dose. If so, irradiation continues in a different area. Quesson et al.^11^, on the other hand, did not focus on the duration, but rather on the intensity of the focused ultrasound irradiation. In addition, they aimed at maintaining a predefined temperature profile. In contrast to Enholm et al.^10^, the calculation of the simulation is performed in real time during the intervention. This is possible by a simplified description of the equation in frequency space. The Fourier transformed equation can be thereby solved with an algebraic expression and the heat radiation amplitude can be found. Together with the MRI-generated heat maps, a control loop can again be implemented. The position of the applicator and tissue or perfusion parameters are determined prior to ablation using reference images and then assumed to be constant during the whole intervention. Nonetheless, control algorithms are also capable of adjusting more than one parameter at a time. Mougenot et al.^12^ transferred the problem of coefficient determination for BHTE to the field of control engineering. They compared the temperature distribution of a HIFU simulation $\theta$ with a sequence of MRI heat maps *T* in a dynamic control loop. The difference between the target temperature *T* and the actual measured temperature $\theta$ was minimized based on the design of a PID controller. This control algorithm takes into account both the current temperature difference and its time derivative, as well as the accumulation of the error over the past time steps. By coupling the controller with the solution of the heat equation in Fourier space, they were able to determine the values of ultrasound absorption, heat diffusion, and perfusion. The optimization with the help of an algorithm for the multidimensional search of the local minimum was carried out by iterative calculation of the heat equation with modified tissue parameters. The evaluation of the approach resulted in a real-time, accurate determination of the parameters with little dependence on noise. These properties lead to a fast termination of the algorithm and therefore to the stability of their method. De Bever et al.^13^ developed another method, where a less complex model is used to describe the heat change. The heat input and removal are described by two simple and flexible exponential equations. Their parameters are immediately updated for targeted voxels based on each new MRI measurement. Thus, any change, no matter the physical background, is taken into account. Because of the constant updating, the predictions of heat distribution need to be accurate only until the next measurement is obtained and not for the entire ablation period.

Finally, methods of inverse problems can be applied to heat propagation and thus to thermal ablation. In the work of Hafid et al.^14^ it was possible to calculate the propagation of temperatures during cryoablation using a few sensors in the tissue. The thermal behavior during the transition from soft to frozen tissue was integrated into the BHTE, which allowed for the prediction of the movement of the cold front. The most relevant thermophysical coefficients of the model could be obtained by inverse evaluation of the temperature sensor data. Verhaart et al.^15^ also worked with point sensor data, two of which are located in different tissue types. Because they developed their approach on patients rather than simulated data, they were able to determine different diffusion and perfusion values for tumor, muscle, and adipose tissue. Their stimulation of an RF hyperthermia could thus be tailored to different patients and sessions. Fuentes et al.^16^ performed in vivo ablations with the support of a BHTE model adapted to MRI temperature data. MRI slices acquired every 5 s made it possible to track the progress of LITT procedures in real time. By optimizing the complex nonlinear perfusion and diffusion terms, the simulation could be adapted to the interventional data.

## Material and methods

**Figure 1:**
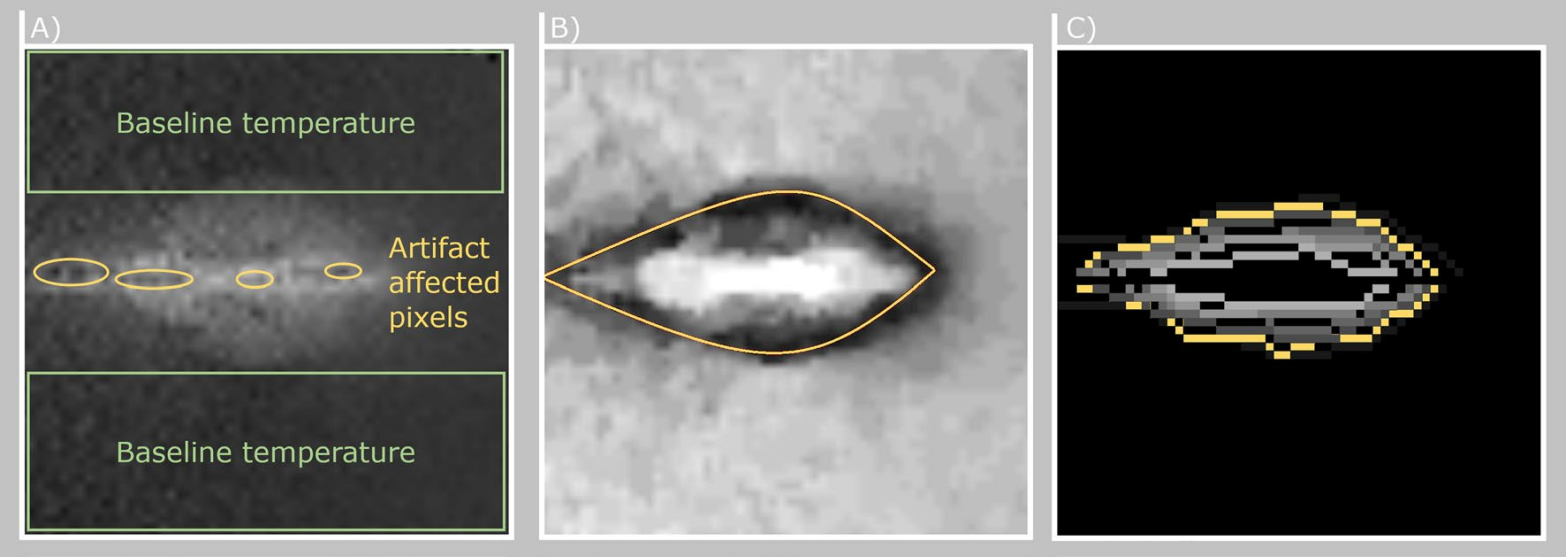
(**A**) Temperature map after computation of the temperature values using the PRFS method. No color-coding was applied for better visibility of baseline temperature (background temperature without any heating) and artifact affected pixels (within and around the needle axis caused by signal cancellation and air). (**B**) Pathfinding map for determining contour line of equal temperature. Darker areas depict regions of low cost for the path finding algorithm. Yellow = Extracted isoline. All values in (**A**) and (**B**) are in the range of [0,100]. (**C**) Eight extracted isotherms within a range of $24\,\,^\circ$C color-coded on a grey-scale. The highlighted yellow isotherm corresponds to $21\,\,^\circ$C.

Many of the related works utilize a mathematical model to simulate the ablation procedures. The prerequisite for clinical use is intervention-specific modeling, which includes both spatial and temporal adjustment of the coefficients. This is because the parameters are not only temperature-dependent and change in the course of the ablation, but different tissue and material types require a separate treatment. In addition, the needle not only acts as an energy source, but its material itself interacts with the emitted heat, which in turn shapes the pattern of heat distribution. Also, more complex geometries of the needle (e.g., in MWA) require special modeling^17^. The consideration of so many physical interactions may lead to complex differential equations with many coefficients. In an interventional setup, these equations must be solved at each time step, and the set of parameters must be optimized regularly. The difficulty here is in reconciling the complexity and the resulting increased accuracy of the computation with a real-time capability.

Therefore, this work aims to reduce the mathematical problem to a diffusion process. Hence, the approach to the modeling of heat distribution shifts from the consideration of a physical optimization problem to the consideration of an optimization problem in computer vision. In the following concept, the goal is not to describe an internal physical process as best as possible, but to extract suitable information from 2D thermometry data in order to map it to a 3D simulation. The measured data thus only adjust the progress of the ablation, while their values are not included in the simulation.

### Isothermal filter

**Figure 2:**
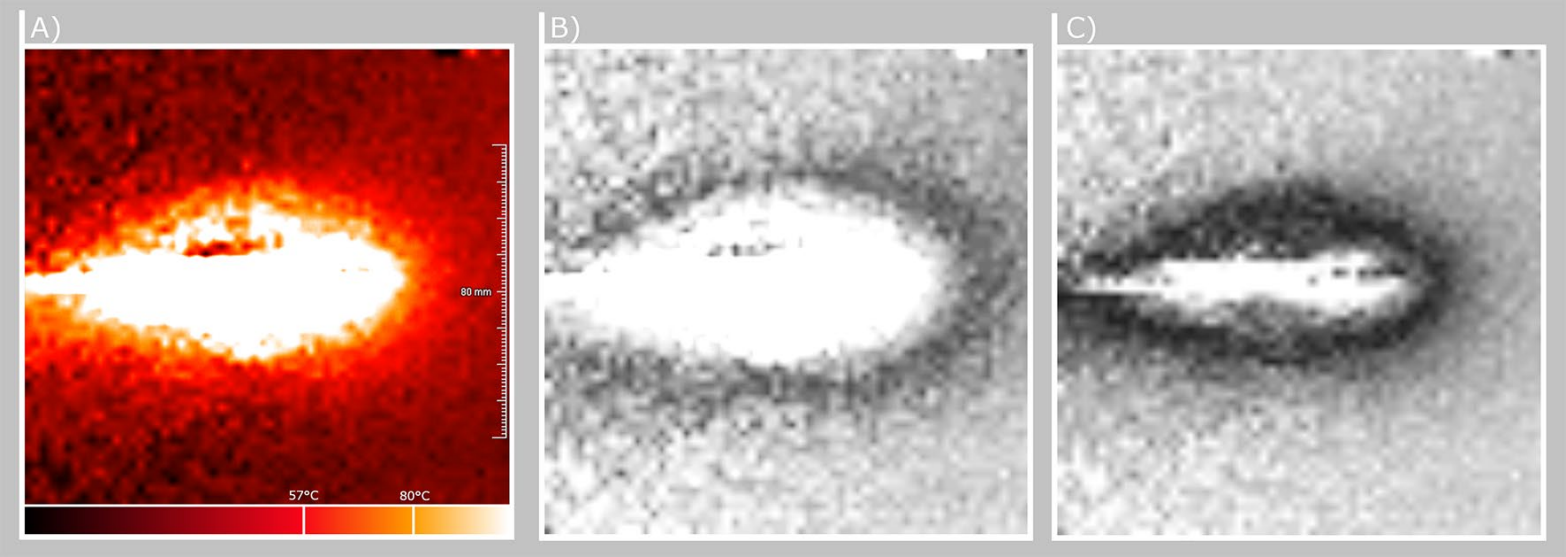
(**A**) Absolute temperature map. (**B**) Relative temperature cost map with a reference value of $25^\circ$C. Darker areas depict lower costs for the path finding algorithm. (**C**) Relative temperature map with a reference value of $35 ^\circ$C. Note how the black outline for lower path finding costs shifted and became more narrow due to the different reference value.

In this work, we utilize our publicly available data set^4^, which consists of thermometry maps rotated around the applicator’s main axis. The data set can be found at Open Science OVGU and consists of single slice 2D GRE images (TE = 3.69 ms, TR = 7.5 ms, flip angle = $7^{\circ }$, FOV = 256 $\times$ 256 mm, matrix = 256 $\times$ 256, bandwidth = 40 Hz/Px, slice thickness = 5 mm). Because of the uniform distribution around the main axis of the applicator the current heat profile can be observed in every image acquired and the location of the applicator artifact (heat source) is always known. Using this information, no mathematical term for a treatment specific heat source needs to be introduced in the equation because the heat profile directly correlates with the heat source distribution. The thermometry maps are characterized by noise and artifacts caused by the applicator. The inter-dependency between the applicator and MRI magnetic field may result in complete erasure or distortion of thermal information around the applicator. Furthermore, the major part of the measured 2D data consists of the baseline temperature of the non heated parts in the phantom (see Fig. 1). To overcome the image corruption problem and to compress the information, the maps are filtered by extracting isotherms. By placing the data in a relative relationship to a reference point, the determination of the isotherm is more robust against noise. This can be done by using Eq. (1):

1 $$\begin{aligned} D_i = |T_i - T_{iso}| \forall i \in N \end{aligned}$$

with $D_i$ referring to the temperature deviation between the temperature isovalue $T_{iso}$ and the measured temperature *T* for all pixel *N* in the image. The idea is that the fluctuations around the isovalue are considered only in the context of the total displacement. By adding up these positive and negative deviations in the calculation of the total distance, the stochastic noise can be eliminated. The global minimization of the Gaussian-distributed noise in the acquired data results in a path that follows temperature of interest. To reduce the complexity of this approach we consider the noise in the image as locally independent and equally occurring for heated and non-heated areas. An example of the relative temperature distribution can be seen in Fig. 2.

The implementation in this work is based on Dijkstra’s algorithm^18^. In the case of the isothermal filter the path can be forced only in the direction of the needle axis. In this way, outliers in the data are also robustly removed. Only directly connected pixels with values close to the thermal isovalue generate a path with low cost.

### Adaptive Pennes’ bioheat simulation

A widely used mathematical model for studying the heat transfer in biological tissue is given by Pennes’ BHTE^19,20^:

2 $$\begin{aligned} \rho (T)c(T)\frac{\partial T(t)}{\partial t} = \underbrace{\nabla (k(T) \nabla T(t))}_{\text {Diffusion Term}} + \underbrace{w_b c_b (T_a - T(t))}_{\text {Perfusion Term}} + Q_m(t) + Q_r(t) \end{aligned}$$

where $\rho$, *c* and *k* are the tissue density, tissue specific heat capacity and tissue thermal conductivity. The perfusion term consists of $w_b$, the blood perfusion rate, $c_b$, the blood specific capacity and $T_a$, the temperature of the arterial blood. $Q_m$ describes the metabolic heat generation rate, $Q_r$ the regional heat source and *T* represents the temperature at a given time point *t*. Regarding the density of blood itself $\rho _b$ there are different approaches. Some authors like Bourantas et al.^21^ treat the blood density individually in the simulation term. Other authors like Zhang et al.^22^ do not report the blood density in their optimization term because it is indirectly included in the blood perfusion rate $w_b$. In the proposed method we follow the example of Zhang et al.^22^ and consider $w_b$ already included in the perfusion rate.

To utilize the Pennes’ BHTE, the position of the heat source must be identified within every acquired 2D image. Because we rotate the images around the applicator’s main axis the heat source must be located on this axis as well. In addition, we break down the simulation problem from a mathematical model to a diffusion process. Therefore, $Q_r(t)$ in Eq. (2) can be set to 0 for all voxel outside the applicator’s main axis. The BHTE, a parabolic partial differential equation, can be physically described as a non-homogeneous heat equation. In addition to the homogeneous part of the diffusion, it consists of positive and negative heat sources, which have no spatial or temporal derivative. By combining and rearranging the terms and coefficients, it can be reduced to the following general equation:

3 $$\begin{aligned} \frac{\partial T(\overrightarrow{\varvec{x}},t)}{\partial t}&= D \cdot \nabla ^2T(\overrightarrow{\varvec{x}},t)+P(\overrightarrow{\varvec{x}},t,T)\nonumber \\ P(x,t,T)&= \frac{w_b c_b (T_a - T(t) + Q_m(t) + Q_r(t))}{\rho (T)c(T)}\nonumber \\ D&= \frac{k}{\rho \cdot c} \end{aligned}$$

Here, *P*(*x*, *t*, *T*) describes the local heat sources and sinks and $\overrightarrow{x}$ refers to the three dimensional point within the volume of interest. For each of the *N* heat sources on a point of the needle axis $r_i$, the orthogonal distance to each of the *M* isotherms $t_m$ is determined and summed up. To obtain the relative temperature distribution along the axis, the total distances are divided by the maximum total distance $q_{max}$ of all *N* points. This results in a relative strength of the heat points $q_i$ in the range [0,1] as given by Eq. (4):

4 $$\begin{aligned} q_i = \frac{1}{q_{max}}\sum ^M_{m = 0}|r_i-t_m| \forall i \in N \end{aligned}$$

By specifying a factor, an absolute distribution can be obtained from the relative distribution. This factor limits the heat input to a maximum temperature and can thus be understood as a vertical shift of the complete temperature distribution. In the form of a newly introduced ablation parameter, $T_{max}$ assigns an absolute value $T_i$ to each $q_i$ using Eq. (5)

5 $$\begin{aligned} T_i = q_i \cdot T_{max} \end{aligned}$$

The quantitative determination of unknown tissue and ablation parameters can be described by the inverse heat conduction problem. In this work, the inverse heat transfer for estimating the values of the simulation parameters is performed using a least-square norm estimation procedure.

**Figure 3:**
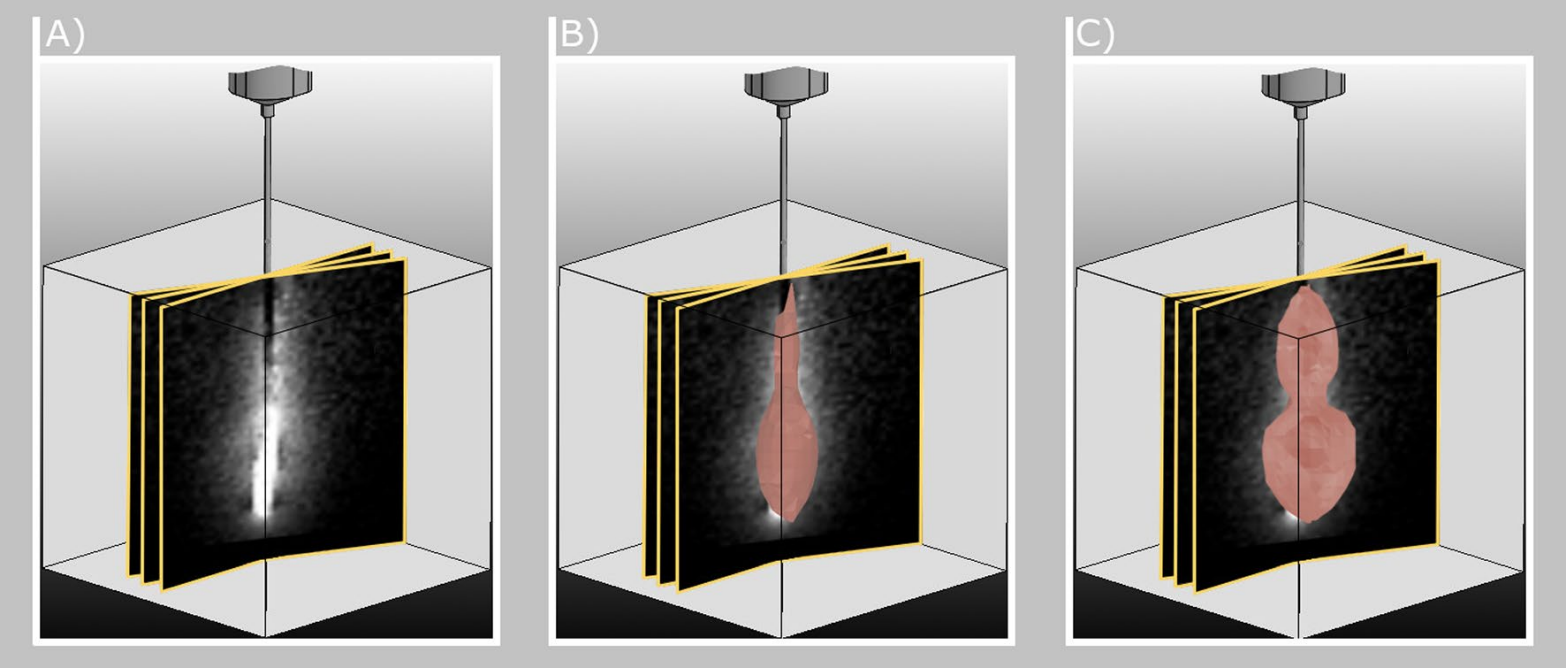
(**A**) Uniformly distributed thermometry maps around the main axis of the applicator. Only three slices are shown as examples. (**B**) 3D simulation (red volume) fitting using a least-square norm estimation (t = 66). (**C**) Final simulation and fitting of the 3D simulation (red volume) (t = 112). Note that the deformation on the left and right side of the simulation is caused by heat sink effects.

The Levenberg-Marquardt algorithm, originally developed for nonlinear parameter estimation problems^23,24^, has been successfully applied to the solution of the ill conditioned inverse heat conduction problem^25,26^. Its combination of steepest descent and the Gauss-Newton method increases robustness and the likelihood for convergence. For optimizing our simulation, the following objective function *f* has to be minimized:

6 $$\begin{aligned} f\left( P^i\right) = \sum ^N_{j = 0} \left[ \overrightarrow{T_E}^i_{j}\left( P^i, \overrightarrow{T_E}^{i-1}_{j}\right) - \overrightarrow{T_{R}}^i_j \right] ^2 \quad \forall t_i \in I_{n} \end{aligned}$$

where $\overrightarrow{T_E}$ is the vector of estimated temperatures at the current discrete time step $t_i$. $\overrightarrow{T_E}$ is obtained by the direct Pennes’ BHTE model. The simulation is based on the state of the optimization at time step $t_{i-1}$ and is corrected by the updated unknown parameter set $P = \{D,T_{max}\}$. $\overrightarrow{T_{R}}$ is the vector of real temperatures extracted from the live 2D thermometry map. The sum squared error between each data point *j* in the live data and the 3D simulation is reduced for each new acquired thermometry map $I_1 \ldots I_n$. An example is shown in Fig. 3.

For defining the time varying tissue temperature *T* for every voxel at every time step *t*, we used Crank-Nicolson’s scheme for finite differences^27^ as in Equation 7.

7 $$\begin{aligned} \frac{T^{i+1}_j - T^i_j}{\Delta t}&= \frac{1}{2} \left( D\frac{T^i_{j+1} - 2T^i_j + T^i_{j-1}}{(\Delta x)^2} + P^i_j + D\frac{T^{i+1}_{j+1} - 2T^{i+1}_j + T^{i+1}_{j-1}}{(\Delta x)^2} + P^{i+1}_j \right) \end{aligned}$$

This method combines the explicit and implicit Euler method in time and central differences in space. Hence, this scheme is unconditionally stable for diffusion equations and has second order spatial and temporal accuracy. For reducing the computational effort to solve the implicit equations in multiple dimensions, we implemented an alternating-direction implicit method^28^. This allows for solving the linear system by only considering tridiagonal matrices, which can be done by the Thomas algorithm. The presented method was implemented on a GPU architecture using the alternating-direction implicit method for parabolic differential equations to further increase the computational speed. All source code is publicly available on GitHub via https://github.com/jalpers/ScientificReports2022_AdaptivePennesSimulation/tree/main.

## Evaluation

### Initial parameter estimation

The initial condition is determined by the baseline temperature before the ablation, with $\varvec{x} = (x,y,z)$ representing the 3D coordinate in the final output volume:

8 $$\begin{aligned} T(\overrightarrow{\varvec{x}},t) = T_0, \end{aligned}$$

The huge amount of unaffected tissue in the peripheral boundary and a constant ambient temperature are suitable for using the Dirichlet boundary condition $\Gamma _E$:

9 $$\begin{aligned} T(\overrightarrow{\varvec{x}},t) = T_{\Gamma }, \quad t>t_0, \overrightarrow{\varvec{x}} \in \Gamma _E \end{aligned}$$

Due to the use of bio protein phantoms, the following parameters are set for the Penne’s BHTE:

10 $$\begin{aligned} Q_m&= 0\text { (no metabolic activity)}\nonumber \\ c_b&= 4182 [\frac{J}{Kg \cdot K}] \nonumber \\ w_b&= \frac{\text {flow rate of pump * density of water}}{r^2\pi \cdot l} \nonumber \\ T_a&= 25 C^{\circ } \end{aligned}$$

The studies summarized by Mohammadi et al^29^ give a range of thermal diffusivity *D* from 0.142 to 3.68 $\frac{\mathrm{mm}^2}{s}$ at 22 $^\circ$C. Considering the increasing values due to temperature dependence, the optimization range is set to [0.1, 5] $\frac{\mathrm{mm}^2}{s}$ with an initial value of $\frac{\mathrm{mm}^2}{s}$. The second parameter to be optimized, $T_{max}$, has an optimization range from $80$ to $300\,\,^\circ$C and starts at the homogeneous and known ambient temperature $T_0$.

### Experimental setup

In this setup, we utilize our publicly available data base for evaluation^4^ . Here, a data base of 13 bio protein phantoms was created according to Bu Lin et al.^30^, and images were acquired by utilizing a 2D GRE sequence (TE = 3.69 ms, TR = 7.5 ms, flip angle = 7$^{\circ }$, FOV = 256 $\times$ 256 mm, matrix = 256 $\times$ 256, bandwidth = 40 Hz/Px, slice thickness = 5 mm) using a 1.5*T* MR scanner (Siemens Avanto, Siemens Healthineers, Erlangen, Germany). Note that the TE/TR are in general too short for good temperature-to-noise ratio but are needed in order to speed up the image acquisition and adjust it to the actual clinical protocol. Here, the images need to be acquired during the after respiratory phase, which limits the maximum acquisition time. Using an optimal TE of roughly 15–20 ms the acquisition time would increase approximately by a factor of 3, which is not suitable in the proposed setup. The acquired images are rotated around the main axis of the applicator, uniformly distributed in eight different orientations. This ensures that the needle artifact is centered in all acquired images. Therefore, the heat profile is comparable for all images and always covers the heat distribution from the heat source to the peripheral ablation margin. The images are used as a sequential input for the algorithm to simulate a live fetching from the MR device, e.g., by using the Siemens Healthineers Access-I Framework for direct scanner control. Because the optimization of the simulation parameters is global and not local, heat sinks caused by vessels are not reflected by the simulation alone. Therefore, we assume that big vessels have been extracted from the pre-clinical data and can be applied to the final simulation outcome to a certain degree.

### Statistical evaluation

For statistical evaluation purpose, the simulated heat map has to be converted in a binary coagulation necrosis. To achieve this, the same thresholds as described in our previous work^4^ and publicly available data sets are applied in the range of $[50,60]\,\, ^\circ$C for each phantom individually (Global threshold).

Regarding the accuracy, we performed a similarity measurement between our simulation and the ground truth using the Dice Score Coefficient (DSC) computed by Eq. (11):

11 $$\begin{aligned} DSC = \frac{2*TP}{2*TP + FP + FN} \end{aligned}$$

taking into account the relation between true positives (TP), false positives (FP) and false negatives (FN). To analyze the error of our results, we used the standard error of the mean (SEM) at a confidence interval level of 95% (p = 0.05) using Eq. (12).

12 $$\begin{aligned} \sigma&= \sqrt{\frac{\sum (x_i-\bar{x})^2}{N-1}}\nonumber \\ SEM&= \frac{\sigma }{\sqrt{N}} * 1.96 \end{aligned}$$

with $\sigma$ = standard deviation, $x_i$ = current sample, $\bar{x}$ = mean value, *N* = sample size and 1.96 = approximated value of the 97.5 percentile of the standard normal distribution. The SEM provides an assumption on how far the sample’s mean is likely to be from the real population mean. In combination with the standard deviation $\sigma$, these statistical values are able to give a decent overview of the performance of our method. We used our publicly available interpolation method as reference to compare to our results^4^.

In addition, we performed tests regarding the robustness of our method. For this purpose, the optimization is performed, on the one hand, with less real-time data, and, on the other hand, with less a priori knowledge about the expected values of the coefficients. The reduction of data can be achieved by not considering thermometry maps from certain orientations. Thus, an experimental setup is generated in which a modified recording protocol is simulated. Reducing the amount of data results in the following configurations: config. 1 = $[0^\circ , 22.5^\circ , 45^\circ , 67.5^\circ ]$, config. 2 = $[90^\circ , 112.5^\circ , 135^\circ , 157^\circ ]$, config. 3 = $[0^\circ , 22.5^\circ ]$, config. 4 = $[90^\circ ]$ and config. 5 = $[22.5^\circ ]$. Additional tests were performed by variation of the optimization parameters initial values and search criteria. Here, the following two configurations were tested: config. 6 = Unrestricted search range with *D* in the range [0.1,1000] and $T_{max}$ also in the range [0.1,1000] and config. 7 = Unrealistic initial parameters with *D* = 10 $\frac{\mathrm{mm}^2}{s}$ $T_{max}$ = $500^\circ C$. All robustness tests were also conducted with an additional threshold configuration (Median). Instead of using the global threshold as explained in our previous work^4^ the best threshold for each individual orientation was identified. Afterwards, the median of these eight thresholds was computed and used for better reflection of the varying conditions inside the tissue.

**Figure 4:**
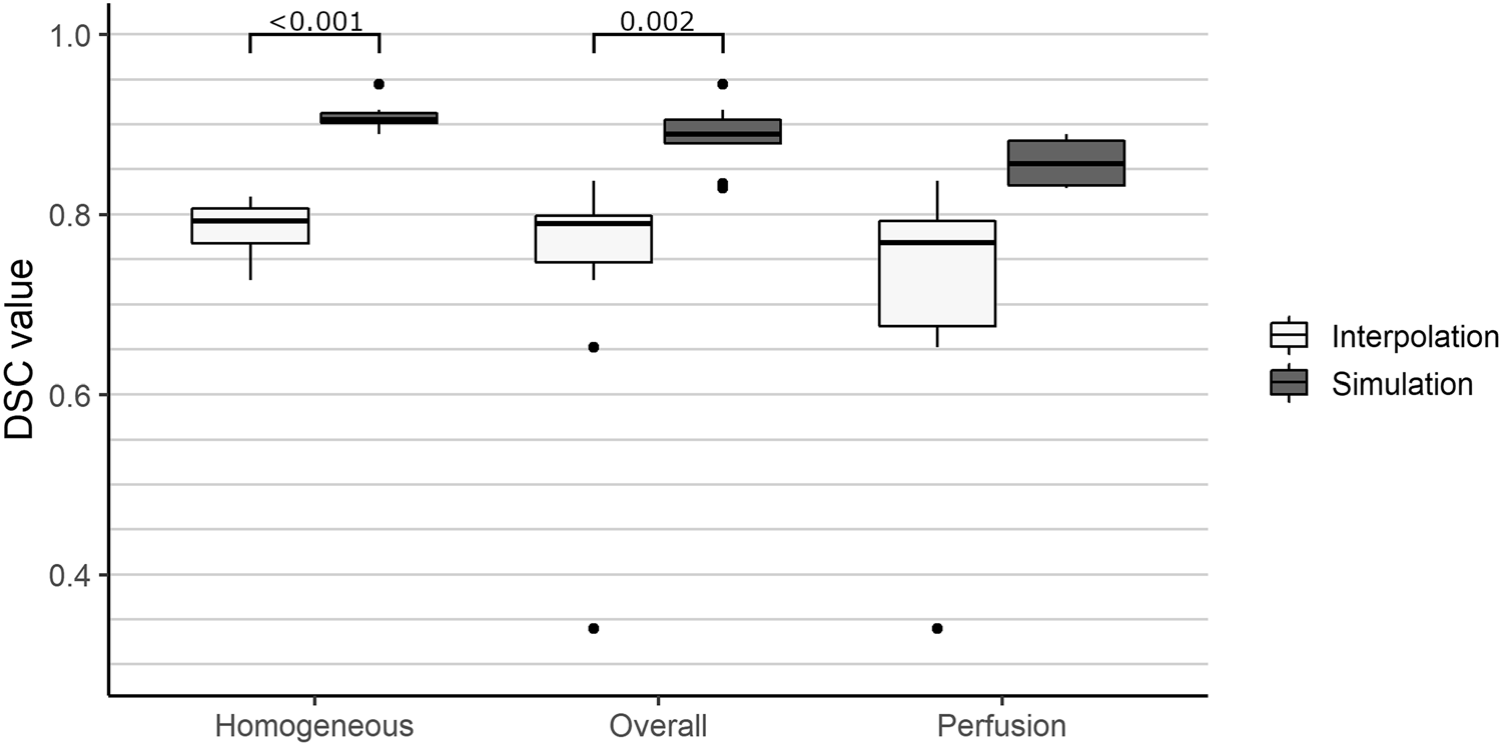
Results of the similarity measurements. The DSC is separated for each phantom category with: Homogeneous = Phantoms without PVC tubes and water perfusion (n = 7). Perfusion = Phantoms with PVC tubes and water perfusion (n = 6). Overall = All phantoms (n = 13). Horizontal lines indicate statistically significant post-hoc pairwise t-test results. No values are located below a DSC of 0.3.

**Figure 5:**
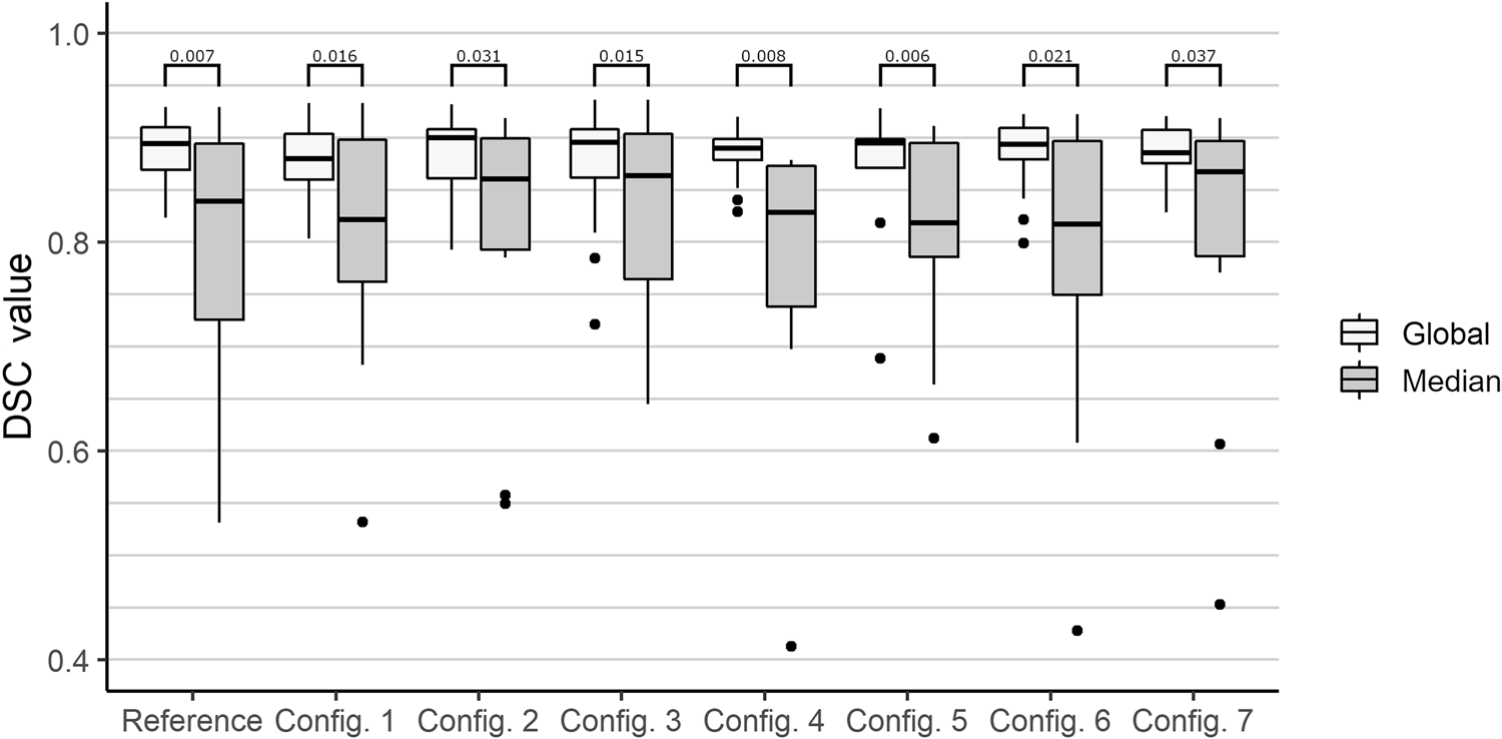
Results of the robustness analysis. Configuration (Config.) 1–5 represent the variation of the number of orientations. Configuration 6 and 7 represent the variation of optimization parameters. Global = globally optimized threshold. Median = Median from the optimized thresholds of each of the eight image orientations. Horizontal lines indicate statistically significant post-hoc pairwise t-test results. No values are located below a DSC of 0.4.

Finally, we performed an ANOVA test paired with post-hoc pairwise t-tests to analyze the significance of each conducted test scenario. All p-values are adjusted using the Bonferroni correction method and reported as horizontal lines in Figs. 4 and 5.

**Table 2:** ANOVAs’ results describing the significance of findings over the test configurations in comparison with our previous method^4^.

Variable	df	F	p	Sig.	$\eta ^2$
**Accuracy**					
Algorithm for all phantoms (overall)	2	23.97	<0.001	*	0.48
Algorithm for perfusion phantoms	2	6.82	0.001	*	0.43
Algorithm for homogeneous phantoms	2	77.69	<0.001	*	0.89
**Robustness**					
Local threshold	84	0.96	0.44		0.02
Global threshold	84	1.03	0.42		0.02
Reference configuration	12	10.38	0.007	*	0.21
Test configuration 1	12	7.86	0.016	*	0.14
Test configuration 2	12	5.95	0.031	*	0.15
Test configuration 3	12	8.12	0.015	*	0.07
Test configuration 4	12	10.09	0.008	*	0.23
Test configuration 5	12	11.14	0.006	*	0.12
Test configuration 6	12	7.05	0.021	*	0.16
Test configuration 7	12	5.5	0.037	*	0.13

The configurations are separated in accuracy and robustness tests. To ensure reproducibility of the p-values the following values are reported: Df = degrees of freedom, F = F-value, p = probability of the data given the null hypothesis, Sig. = p-values less than the traditional $\alpha$ <0.05 are marked with a “*”, $\eta ^2$ = Generalized Eta-Squared measure of effect size.

## Results

ANOVA test results can be seen in Table 2. Evaluation results regarding the similarity and the corresponding post-hoc results can be observed in Fig. 4. Observations show an average overall DSC of 0.89±0.04 exceeding our previous temperature interpolation approach^4^ by a total of 0.10 regarding the overall average DSC. With respect to the homogeneous and perfusion phantoms separately, we can observe an average DSC of 0.91 ± 0.02 and 0.86 ± 0.03, respectively. The SEM values are constant at 0.01 for all three groups. Regarding the standard deviation, our initial method shows values of 0.3 and 0.17 for the homogeneous and perfusion phantoms, respectively. The simulation approach presented in this work shows a standard deviation of 0.02 and 0.03 for the homogeneous and perfusion phantoms, respectively. In conclusion, the proposed method does not only exceed our previous version by a mean DSC of 0.10, but it is also more robust towards corrupted images, which caused the temperature interpolation method to fail.

With respect to the robustness tests, we are able to observe slight differences in the overall mean DSC for all phantoms, but we were not able to detect any significant changes between the different configurations. Therefore, we can assume that the number of different orientations during image acquisition, as well as the initial parameter values and boundary conditions, do not influence the performance of our method. Regarding the variation of the threshold configuration we can observe significant differences between the global and median approach. All results can be observed in Fig. 5 including the post-hoc pairwise t-test results as horizontal lines.

All results were generated on a Desktop PC (Intel Core i7-2600K, 16GB RAM, NVIDIA GeForce GTX 1060 with 6GB memory) with GPU acceleration and computation of the parameters *D* and $T_{max}$ took $1.05 \pm 0.26$ s for each newly acquired image. Computation times are based on a volume size of 60 $\times$ 60 $\times$ 60 voxels and 100 repetitions.

## Discussion and conclusion

One of the advantages of the proposed method is the rotation of the acquired images around the main axis of the applicator. This results in a typical shape of heat propagation, even when vessels causing a heat sink effect are present. Hence, the measurement can be easily verified and corrected. In addition, every image contains the heat source in the form of the elongated artifact of the applicator. This artifact can be detected and traced back to find the source of the heat distribution used for simulation. Nonetheless, the preparation of this sequence protocol can be troublesome for inexperienced MRI users. In order to work as accurately as possible, the rotated images should intersect in the center of the field of view, and this intersection should correspond to the applicator’s main axis. Even though this should not be troublesome for an experienced MRI user, it might be cumbersome for new radiologists or medical technical assistants. Another problem may arise from out-of-plane angulation or bending of the needle. The more the needle is away from the intersection of the rotated planes, the less accurate the simulation method will be because the simulated applicator axis (heat source) is inaccurate. Here, a possible solution could be the inclusion of a priori knowledge about the applicator’s position, either from manual annotation or planning. Additionally, an automated slice positioning algorithm can be used to find the optimum positioning of the images during intervention. Van der Kouwe et al.^31^ introduced an atlas-based approach for aligning the MRI plane in the region of interest. This approach may be applied to our simulation by acquisition of a 3D reference volume after needle insertion and prior treatment. The needle artifact can be extracted automatically, and an optimal MRI plane position can be computed and applied. In addition, to the automated definition of plane orientation the restrictions regarding the sequence parameters need more attention. The used TE of 3.69 ms is not optimal regarding the temperature-to-noise ratio. Here, a proper study has to be conducted to identify the best trade off between image acquisition time and temperature-to-noise ratio in order to optimize the setup. Another problem with our approach arises from the used bio protein heat phantoms. According to Bu Lin et al.^30^, the coagulation of the phantom highly depends on the pH-value, which can vary within the phantom itself. For this reason, the computation of the coagulation necrosis using, e.g., a threshold approach, will introduce an error of unknown size. In addition, the used PVC tubes to simulate a heat sink effect do not provide real tissue dependent parameters. Hence, the approximation of the perfusion term does not reflect the real heat distribution. This problem can be addressed by evaluation of the method in a more clinical context. Here, perfused ex vivo porcine livers can be used, as introduced by Becker et al.^32^, to create a more realistic data base. Regarding the algorithm, we used a global approach for optimization of the parameters. This leads to a more or less homogeneous prediction of the ablation zone, but does not take into account local tissue variation or heat sinks without a priori knowledge based on e.g., segmentation of structures. Here, it would be suitable to look for a computationally efficient solution for local optimization of the parameters. This approach could be combined with our previous method. The local parameters could be optimized for one image locally and then interpolated between the other acquired images. In this context, the image noise should also be analyzed regarding local dependencies especially in regions around the applicator’s main axis. Lastly, it is important to not just compare our results with other 2D–3D reconstruction methods but also to other full 3D thermometry approaches. Especially the work of Zhang et al.^33^ provide promising results and could be considered for a in depth comparison study.

### Conclusion

We proposed a new approach for an adaptive Penne’s BHTE for interventional MR-guided tumor ablation. Our approach is robust towards outliers and artifacts and shows promising results of up to 90% similarity to a manually extracted ground truth. Due to the unique image acquisition protocol, we are able to identify the heat source in every image and therefore are not limited to a specific heat source term. This reduces the computational effort and allows the method to be applicable to a wide range of clinical setups. Future work should focus on a local optimization of the simulation parameters instead of a global optimization. We believe that this approach would be able to accurately detect heat sinks in the data. In summary, our method shows a high potential to aid the performing radiologist during minimally-invasive thermal procedures and increase the success rate, while not necessarily hampering the workflow of the individual clinician.

## Data Availability

The data sets processed and analysed during the current study are available in the Open Science Repository for Research Data and Publications of OVGU (Creative Common License 4.0), Open Science OVGU. In addition, the data sets generated during and/or analysed during the current study are available from the corresponding author on reasonable request. The source code used for generating the results presented in this study are publicly available via https://github.com/jalpers/ScientificReports2022_AdaptivePennesSimulation/tree/main.

## References

[1] Rieke V (2011). Mr thermometry. Intervent. Magn. Reson. Imaging.

[2] Laimer G (2020). Minimal ablative margin (mam) assessment with image fusion: An independent predictor for local tumor progression in hepatocellular carcinoma after stereotactic radiofrequency ablation. Eur. Radiol..

[3] de Senneville BD, Coupé P, Ries M, Facq L, Moonen CT (2021). Deep correction of breathing-related artifacts in real-time mr-thermometry. Comput. Med. Imaging Graph..

[4] Alpers, J. *et al.* 2.5 d thermometry maps for mri-guided tumor ablation. In *International Conference on Medical Image Computing and Computer-Assisted Intervention* 311–320 (Springer, 2021). 10.1007/978-3-030-87202-1_30.

[5] Johnson PC, Saidel GM (2002). Thermal model for fast simulation during magnetic resonance imaging guidance of radio frequency tumor ablation. Ann. Biomed. Eng..

[6] Roujol, S., de Senneville, B. D., Hey, S., Moonen, C. & Ries, M. Extended kalman filtering for mr-thermometry guided high intensity focused ultrasound using the bio heat transfer equation. In *2011 18th IEEE International Conference on Image Processing* 2281–2284 (IEEE, 2011). 10.1109/ICIP.2011.6116094.

[7] Todd N, Payne A, Parker DL (2010). Model predictive filtering for improved temporal resolution in MRI temperature imaging. Magn. Reson. Med..

[8] Fuentes D, Yung J, Hazle JD, Weinberg JS, Stafford RJ (2011). Kalman filtered mr temperature imaging for laser induced thermal therapies. IEEE Trans. Med. Imaging.

[9] De Senneville BD, Roujol S, Hey S, Moonen C, Ries M (2012). Extended kalman filtering for continuous volumetric mr-temperature imaging. IEEE Trans. Med. Imaging.

[10] Enholm JK (2009). Improved volumetric mr-hifu ablation by robust binary feedback control. IEEE Trans. Biomed. Eng..

[11] Quesson B, Vimeux F, Salomir R, de Zwart JA, Moonen CT (2002). Automatic control of hyperthermic therapy based on real-time fourier analysis of mr temperature maps. Magn. Reson. Med..

[12] Mougenot, C., Kabongo, L., Quesson, B. & Moonen, C. T. Mrghifu: Feedback temperature control with automatic deduction of bht tissue parameters. In *AIP Conference Proceedings, vol. 1113* 231–235 (American Institute of Physics, 2009). 10.1063/1.3131419.

[13] de Bever J, Todd N, Payne A, Christensen DA, Roemer RB (2014). Adaptive model-predictive controller for magnetic resonance guided focused ultrasound therapy. Int. J. Hyperthermia.

[14] Hafid M, Lacroix M (2017). Fast inverse prediction of the freezing front in cryosurgery. J. Therm. Biol..

[15] Verhaart RF (2015). Accurate 3d temperature dosimetry during hyperthermia therapy by combining invasive measurements and patient-specific simulations. Int. J. Hyperthermia.

[16] Fuentes D (2010). Adaptive real-time bioheat transfer models for computer-driven mr-guided laser induced thermal therapy. IEEE Trans. Biomed. Eng..

[17] Deshazer G, Prakash P, Merck D, Haemmerich D (2017). Experimental measurement of microwave ablation heating pattern and comparison to computer simulations. Int. J. Hyperthermia.

[18] Dijkstra EW (1959). A note on two problems in connexion with graphs. Numer. Math..

[19] Pennes HH (1948). Analysis of tissue and arterial blood temperatures in the resting human forearm. J. Appl. Physiol..

[20] Wissler EH (1998). Pennes’ 1948 paper revisited. J. Appl. Physiol..

[21] Bourantas, G. C., Joldes, G. R., Wittek, A. & Miller, K. A flux-conservative finite difference scheme for the numerical solution of the nonlinear bioheat equation. In *Computational Biomechanics for Medicine* 69–81 (Springer, 2019). 10.1007/978-3-319-75589-2_7.

[22] Zhang J, Chauhan S (2019). Real-time computation of bio-heat transfer in the fast explicit dynamics finite element algorithm (fed-fem) framework. Numer. Heat Transfer Part B Fund..

[23] Levenberg K (1944). A method for the solution of certain non-linear problems in least squares. Q. Appl. Math..

[24] Marquardt DW (1963). An algorithm for least-squares estimation of nonlinear parameters. J. Soc. Ind. Appl. Math..

[25] Min T, Chen X, Sun Y, Huang Q (2014). A numerical approach to solving an inverse heat conduction problem using the levenberg-marquardt algorithm. Math. Probl. Eng..

[26] Cui M, Zhao Y, Xu B, Gao X.-w (2017). A new approach for determining damping factors in levenberg-marquardt algorithm for solving an inverse heat conduction problem. Int. J. Heat Mass Transfer.

[27] Crank, J. & Nicolson, P. A practical method for numerical evaluation of solutions of partial differential equations of the heat-conduction type. In *Mathematical Proceedings of the Cambridge Philosophical Society, vol. 43* 50–67 (Cambridge University Press, 1947). 10.1017/S0305004100023197.

[28] Douglas J, Gunn JE (1964). A general formulation of alternating direction methods. Numer. Math..

[29] Mohammadi A, Bianchi L, Asadi S, Saccomandi P (2021). Measurement of ex vivo liver, brain and pancreas thermal properties as function of temperature. Sensors.

[30] Bu-Lin Z (2008). A polyacrylamide gel phantom for radiofrequency ablation. Int. J. Hyperthermia.

[31] van der Kouwe AJ (2005). On-line automatic slice positioning for brain mr imaging. Neuroimage.

[32] Becker D (2019). Model assisted analysis of the hepatic arterial buffer response during ex vivo porcine liver perfusion. IEEE Trans. Biomed. Eng..

[33] Zhang L, Armstrong T, Li X, Wu HH (2019). A variable flip angle golden-angle-ordered 3d stack-of-radial mri technique for simultaneous proton resonant frequency shift and t1-based thermometry. Magn. Reson. Med..

